# Streamlining Preparedness: A Practical Pathway to Special Pathogens Management

**DOI:** 10.3390/tropicalmed10030072

**Published:** 2025-03-11

**Authors:** Sarah Irene Brown, Priya Dhagat, Aishani V. Aatresh, Saoirse Bodnar, Syra Madad

**Affiliations:** 1 System-Wide Special Pathogens Program, New York City Health and Hospitals, New York, NY 10004, USA; dhagatp@nychhc.org (P.D.); aishani@alumni.harvard.edu (A.V.A.); sbodnar@alumni.princeton.edu (S.B.); syra.madad@nychhc.org (S.M.); 2 Department of Global Health, Princeton University, Princeton, NJ 08544, USA; 3 Program on Science, Technology, and Society, Harvard University, Cambridge, MA 02138, USA

**Keywords:** preparedness, high consequence infectious disease, exercise, protocol, emergency management, emergency preparedness, special pathogens

## Abstract

Managing special pathogens cases, also known as high consequence infectious diseases, presents unique challenges for healthcare systems. It requires thorough planning and comprehensive operational protocols, as well as an appreciation of how human and organizational factors influence readiness. Based on the outcomes from a full-scale Ebola Virus Disease exercise at New York City Health and Hospitals (NYC Health + Hospitals), this paper presents a checklist of considerations to promote healthcare facility preparedness for special pathogens and to minimize gaps between protocol design and real-world implementation. This approach not only strengthens compliance with the new Joint Commission requirements but also provides a replicable framework for enhancing special pathogens preparedness within other healthcare systems.

## 1. Introduction

Patients infected with special pathogens (SPs)—highly infectious agents that can cause severe illness—may appear in healthcare systems at any time and through any entry point [1]. Recent outbreaks, including Lassa fever in Nigeria [2], Marburg in Tanzania [3], and H5N1 in countries around the world [4], along with imported cases of Ebola and Lassa fever in the UK and US [5,6], underscore the critical need for robust hospital preparedness. The fatality rates of these diseases can be as high as 88%, demonstrating the danger of unconstrained spread [7]. In 2024, infection prevention and control measures for special pathogens became a requirement for hospitals by the Joint Commission (TJC), a major accreditation body for healthcare organizations across the United States [8,9].

Managing patients suspected to have a special pathogen infection poses unique challenges for healthcare systems, and the granular steps required to build and maintain readiness are often unclear [9,10,11]. Significant gaps in preparedness can arise from a disconnect between public health guidance and frontline healthcare expectations or operations (screening, isolation, patient care capabilities, waste management, and patient transport). For example, protocol seldom anticipates the human and organizational complexities that muddle preparedness in real life scenarios [12]. Fear, information overload, communication challenges, and unfamiliar workflows can make it challenging to put protocol to practice [13,14].

To evaluate the effectiveness of written preparedness and response plans in clinical settings, the New York City Health and Hospitals System-wide Special Pathogens Program conducted a full-scale exercise (FSE) focused on the Identify, Isolate, and Inform (III) approach, waste management, and infection control measures. FSEs include multiple agencies and disciplines, and simulate real events as closely as possible. The exercise revealed gaps in preparedness, prompting the development of a comprehensive Special Pathogens Readiness Checklist—a tool that acknowledges real-world complexities and integrates them into practical planning considerations. The tool is included in this article for customization and implementation by other healthcare facilities.

## 2. Methods

### Exercise Design

In 2021, the System-wide Special Pathogens team conducted a full-scale exercise (FSE) focused on Ebola virus disease to assess the healthcare system’s capacity to manage a case with one of the highest levels of transmissibility and stringent waste precautions. The exercise was especially timely, following the Ebola virus disease outbreak in the Democratic Republic of the Congo in 2020 [15] and an outbreak in Guinea in early 2021 [16]. The NYC Health + Hospitals Ebola virus disease FSE involved all actors who would be engaged in the event of a true suspected SP patient, including multiple frontline acute care hospitals, the local health department, the fire department, a public health lab, and a waste management vendor. Frontline hospitals handle screening, identification, and clinical care. Suspected SP cases are reported to the local health department, initiating bidirectional communication. The guidance at the time dictated that the fire department assist in transferring suspected SP patients from frontline hospitals to the Regional Emerging Special Pathogen Treatment Center, a facility specifically prepared to handle highly infectious diseases. The public health lab analyzes patient specimens to identify the disease in question. Lastly, the waste management vendor ensures the safe handling of Category A waste—infectious waste that holds the potential to cause serious illness or death. The exercise included all relevant actors in order to best determine gaps between protocol and lived experience.

This FSE assessed hospitals’ abilities pertaining to the following objectives:

- Safely follow infectious diseases screening and identification protocols.
- Promptly isolate the patient.
- Don and doff personal protective equipment.
- Notify the local health department, internal response teams and department leadership.
- Follow intra-facility patient transfer plans.
- Follow waste management plans.

Patient actors with symptoms, travel history, and epidemiological risk factors for Ebola virus infection presented to four emergency departments and, as the exercise progressed, documented the outcomes. A total of seven hospitals and agencies across four New York City boroughs participated in the FSE. Following the exercise, facility-specific and system-wide debriefs were conducted. Successes, challenges, and areas for improvement were documented and analyzed.

## 3. Results

Common challenges emerged across the frontline hospitals, including delays in travel and symptom screening, inability to promptly isolate the patient, overlooked engineering controls, and unexpected situations that tested administrative measures, which proved that there was room for systematic improvement to more comprehensively account for human and organizational factors which could improve a state of readiness. Task tracking and simplifying workflows, especially for rare scenarios such as Ebola, emerged as considerations for improvement, which are consistent with the literature [17,18].

Table 1 highlights exercise themes and identifies key considerations for improvement.

## 4. Discussion

The following 10 domains are included in the checklist:

1. Identification and Routine Screening
2. Engineering Controls
3. Administrative Controls
4. Isolation
5. Staff Safety/PPE
6. Patient Assessment and Management
7. Laboratory Testing
8. Notification and Activation of HICS
9. Cleaning, Disinfection, and Spills and Waste Management
10. Inter-facility Transfer

Myriad observations during FSE debriefs, along with guidance from the CDC and NYS DOH, informed the development of a Special Pathogens Readiness Checklist, which is composed of an array of planning considerations [19,20,21,22,23,24,25]. The tool combines FSE-derived knowledge of real-world complexities, feedback from healthcare providers across clinical departments, and the latest literature on how to effectively prepare for infectious disease threats [8,18,19,20,21]. The tool aligns with guidance from the CDC on Viral hemorrhagic fevers (VHFs), a category to which EVD belongs, including the CDC’s Infection Control Guidance, Guidance for Personal Protective Equipment, Clinical Testing and Screening, Evaluating an Ill Person for VHF, Handling VHF-Associated Waste, Interim Guidance for Environmental Infection Control in Hospitals, and Infection Presentation and Control Recommendations for Patients in U.S. Hospitals who are Suspected or Confirmed to have Selected Viral Hemorrhagic Fevers [26]. This is not to suggest that the checklist encompasses all of the specific details in each guidance document but rather that it serves as a cohesive and organized framework to guide readers through the key action items.

To supplement key public health guidelines, the SP Readiness Checklist includes the four categories of readiness identified through the FSE and their respective considerations for process improvement, as described in the results. These items serve as examples of how the team utilized full-scale exercises to identify gaps between protocol and practice, and then built upon these learnings to improve larger processes. The redundant screening protocol reminders within the Identification and Routine Screening category of readiness were included in Section 1, boxes 1, 3, and 4 of the appendix (Appendix A). The alternative modes of communication within the Engineering Controls category of readiness were included in Section 2, box 4, and Section 6, box 3 of the appendix (Appendix A). The workflow to manage the hallways and the clarification of the role of hospital security within the Administrative Controls readiness category were included in Section 3, box 2, and Section 4, boxes 1 and 3 of the appendix (Appendix A). The list of critical tasks that must be completed prior to isolating the patients within the Isolation category of readiness were included in the SP Readiness Checklist Section 4, boxes 4, 5, and 6 of the appendix (Appendix A). In this manner, key observations and lessons learned from dozens of exercises were incorporated into the checklist in order to better align guidance with lived scenarios.

The Special Pathogens Readiness Checklist highlights considerations for ongoing preparedness and may be utilized in anticipation of patients presenting to a facility with a suspected or confirmed special pathogen infection. It uses plain language, allows staff to check off completed tasks and add relevant notes to the master document, and consolidates considerations into one document (Appendix A). It should be adapted based on the healthcare facility’s risk assessment, local regulations, and capabilities of the health department. The complete checklist is included as in Appendix A.

While we believe that this tool will be useful for other healthcare systems, there are several limitations of the SP Readiness Checklist and of its associated research to date. First, the team only had access to qualitative observations. Quantitative assessments of the extent to which use of the checklist improves hospital readiness for special pathogens are important to assess its efficacy. Further, it is possible that FSEs diverge from true special pathogen cases in process considerations. However, the limited number of special pathogens outbreaks within the United States makes it challenging to assess hospital readiness in non-exercise scenarios. Lastly, the Special Pathogens Readiness Checklist was developed for the NYC public hospital system and further research could be helpful to better incorporate the particularities of disparate care settings.

## 5. Conclusions

Proactive planning is essential for the prompt identification and effective management of special pathogens cases. With an increasing frequency of special pathogens outbreaks and travel-associated cases, the need for preparedness is ubiquitous worldwide [27,28]. Protocols, encoded in neat and distilled documents, only act as scaffolding. Human, organizational, and infrastructure factors influence the state of preparedness for healthcare facilities, and must be incorporated into plans and processes. Bridging expectations and realistic operational capabilities through exercises and planning tools, such as the Special Pathogens Readiness Checklist, is critical to provide the necessary structural integrity for a true state of preparedness.

The Special Pathogens Readiness Checklist presented in this paper serves as a tool to improve healthcare facility preparedness and may be used assist with compliance with TJC requirements. Using FSEs to move from the theoretical to the lived, the checklist manages and leverages key human and organizational factors, enables seemingly disparate factors to be considered in tandem, facilitates collective reflection to guide constructive action, and acknowledges that all stakeholders involved play a crucial role in safeguarding healthcare workers and public health. In order to remain attentive as a healthcare system, country, and world, we must embrace the intrinsic human nature that accompanies our work to sharpen our response to whichever pathogen may be around the corner.

## Figures and Tables

**Table 1:** Exercise Findings and Improvement Points.

Categories of Readiness	Exercise Outcomes/Themes	Considerations for Process Improvement
**Identification and Routine Screening**	Delayed or incomplete travel and symptom screening.	Add redundant screening protocol reminders within the electronic medical record as well as signage in registration and triage areas.
**Engineering Controls**	Communication barriers between healthcare workers inside of the isolation room and support staff outside of the isolation.	Utilize alternative methods for communication, such as intercom, telephone or video services.
**Administrative Controls**	Difficulties with patient transfer (e.g., clearing the hallway when patient is being moved while preventing disruptions to normal operations).	Develop a protocol and conduct training for efficient communication to hospital security and a simple workflow to best support management of hallways if patient transfer is warranted while simultaneously preventing disruption of the emergency department.
**Isolation**	Challenges with removal of medical equipment/unnecessary supply from the isolation room and prompt placement of required supply.	Develop a simple list of critical tasks to be performed prior to isolating the patient, such as removing any medical equipment from the room and ensuring PPE and supplies are available.

**Figure A1:**
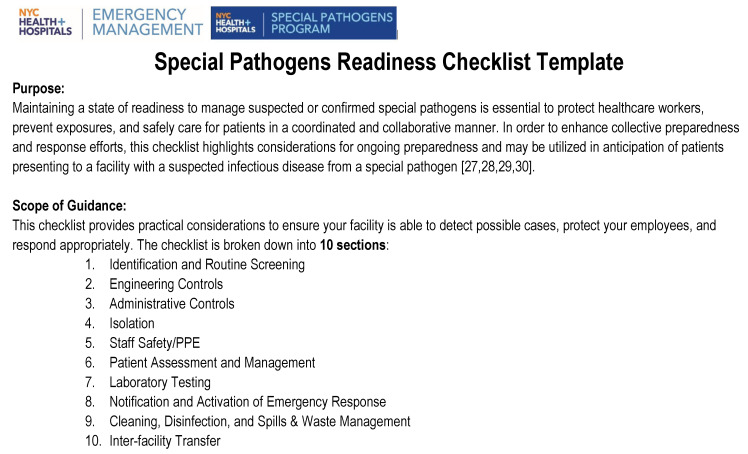

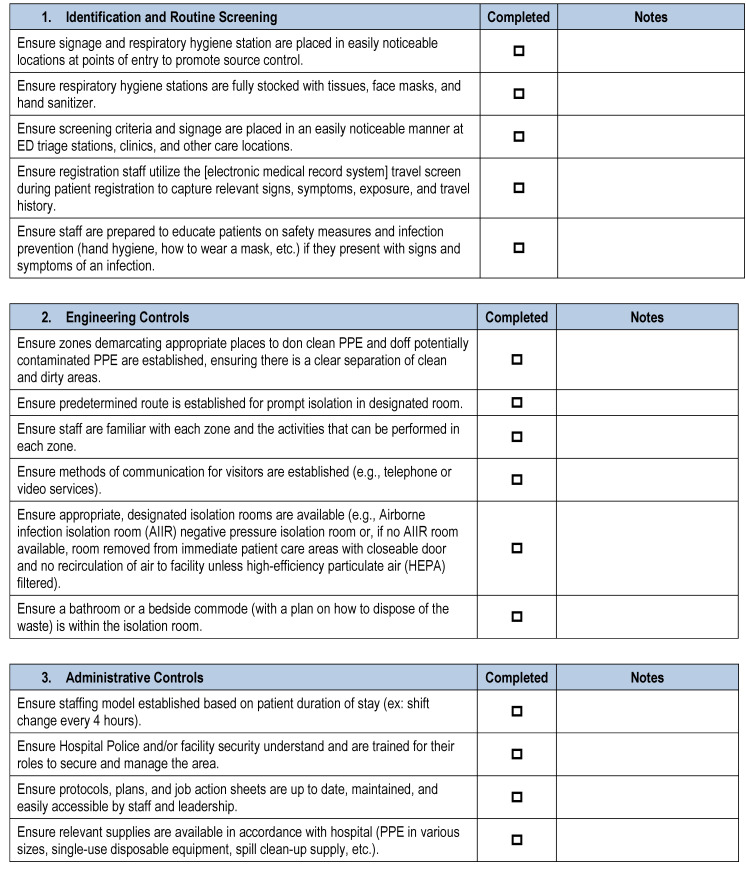

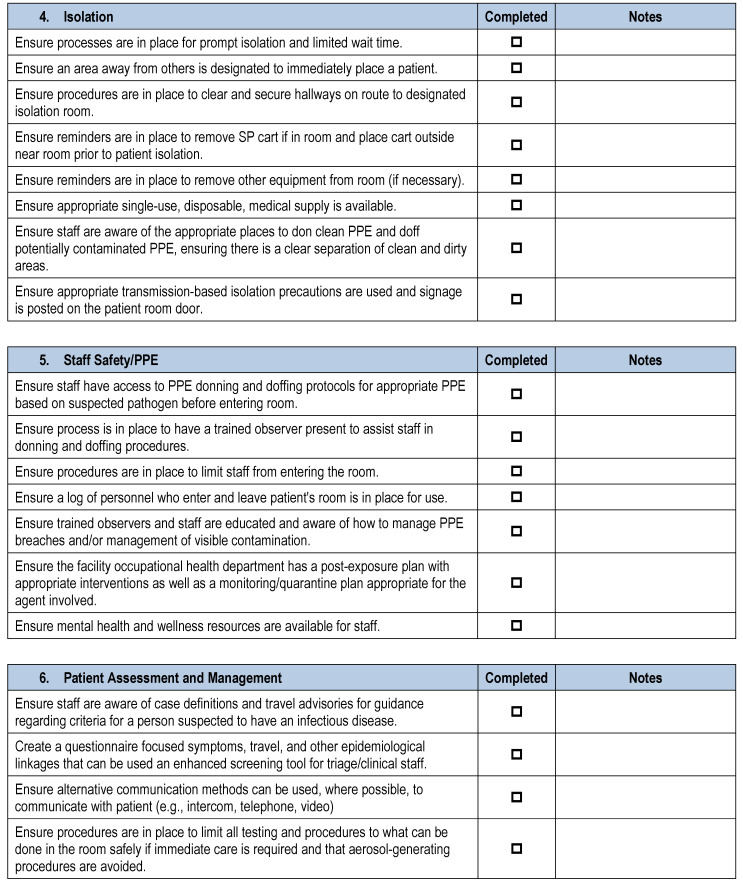

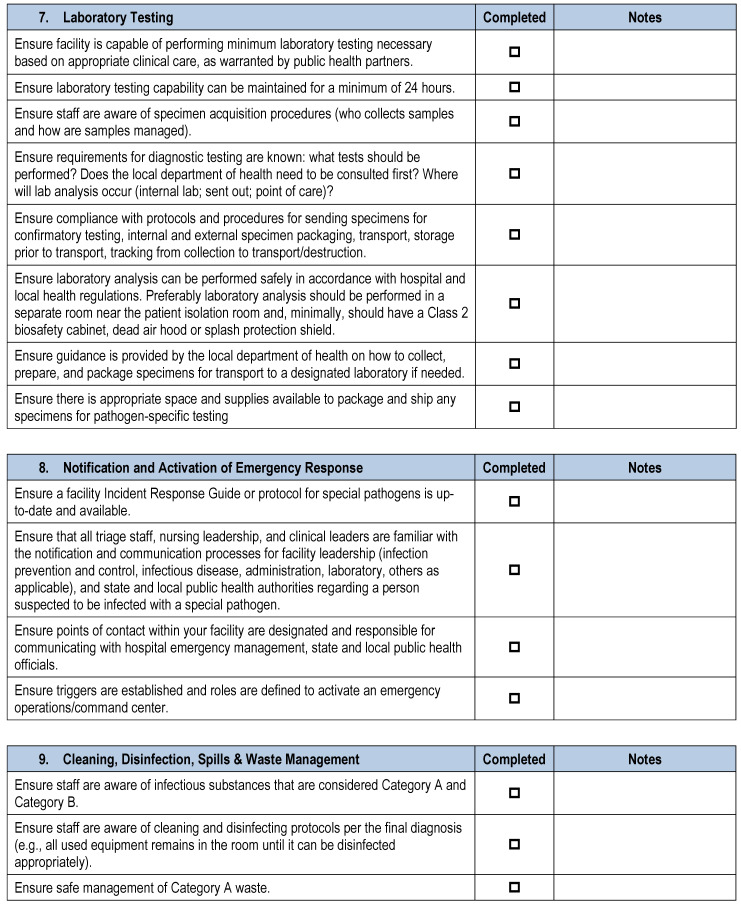

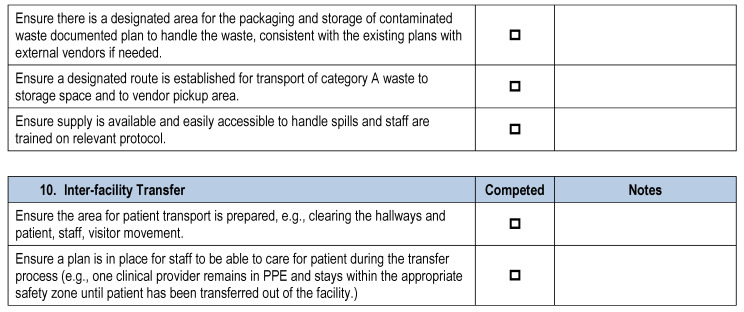
Special Pathogens Readiness Checklist Template [27,28,29,30].

## Data Availability

The qualitative observations presented in this study are included in the article. Further inquiries can be directed to the corresponding author.

## References

[1] NETECNSPS The NETEC Survival Guide–Preparing for Ebola & Other Special Pathogens. NETEC. 21 December 2018. https://netec.org/2018/12/21/netec-survival-guide/.

[2] CDC Lassa Fever Situation Report Epi Week 2: 6th–12th January 2025-Nigeria ReliefWeb. 12 January 2025. https://reliefweb.int/report/nigeria/ncdc-lassa-fever-situation-report-epi-week-2-6th-12th-january-2025.

[3] Outbreak of Suspected Marburg Virus Disease–United Republic of Tanzania. https://www.who.int/emergencies/disease-outbreak-news/item/2025-DON552.

[4] Avian Influenza. https://www.who.int/westernpacific/wpro-emergencies/surveillance/avian-influenza.

[5] CDC (2024). Lassa Fever Suspected in Death of U.S. Traveler Returning from West Africa. CDC Newsroom. https://www.cdc.gov/media/releases/2024/s1028-lassa-fever.html.

[6] Reece S., Brown C.S., Dunning J., Chand M.A., Zambon M.C., Jacobs M. (2017). The UK’s multidisciplinary response to an Ebola epidemic. Clin. Med..

[7] Marburg Virus Disease. https://www.who.int/news-room/fact-sheets/detail/marburg-virus-disease.

[8] R3 Report Issue 41: New and Revised Requirements for Infection Prevention and Control for Critical Access Hospitals and Hospitals. https://www.jointcommission.org/standards/r3-report/r3-report-issue-41-new-and-revised-requirements-for-infection-prevention-and-control-for/.

[9] Sauer L.M., Mukherjee V., on behalf of the NETEC Leadership Team (2022). Special Pathogens Readiness in the United States: From Ebola to COVID-19 to Disease X and Beyond. Health Secur..

[10] A New Species of Trouble Pandemic Center School of Public Health Brown University. https://pandemics.sph.brown.edu/new-species-trouble.

[11] A Nation Unprepared: Incomplete Implementation of the National Blueprint for Biodefense. Bipartisan Commission on Biodefense. https://biodefensecommission.org/events/a-nation-unprepared-incomplete-implementation-of-the-national-blueprint-for-biodefense/.

[12] Fu L., Wang X., Wang D., Griffin M.A., Li P. (2020). Human and organizational factors within the public sectors for the prevention and control of epidemic. Saf. Sci..

[13] Meyer D., Sell T.K., Schoch-Spana M., Shearer M.P., Chandler H., Thomas E., Rose D.A., Carbone E.G., Toner E. (2018). Lessons from the domestic Ebola response: Improving health care system resilience to high consequence infectious diseases. Am. J. Infect. Control.

[14] Pennathur P.R., Herwaldt L.A. (2017). Role of Human Factors Engineering in Infection Prevention: Gaps and Opportunities. Curr. Treat. Options Infect. Dis..

[15] CDC (2024). About Viral Hemorrhagic Fevers. Viral Hemorrhagic Fevers (VHFs). https://www.cdc.gov/viral-hemorrhagic-fevers/about/index.html.

[16] Ebola Outbreak 2021-N’Zerekore, Guinea. https://www.who.int/emergencies/situations/ebola-2021-nzerekore-guinea.

[17] Houghton C., Meskell P., Delaney H., Smalle M., Glenton C., Booth A., Chan X.H.S., Devane D., Biesty L.M. Barriers and Facilitators to Healthcare Workers’ Adherence with Infection Prevention and Control (IPC) Guidelines for Respiratory Infectious Diseases: A rapid qualitative evidence synthesis-Houghton, C-2020 Cochrane Library. https://www.cochranelibrary.com/cdsr/doi/10.1002/14651858.CD013582/full.

[18] Sayfi S., Charide R., Elliott S.A., Hartling L., Munan M., Stallwood L., Butcher N.J., Richards D.P., Mathew J.L., Suvada J. (2024). A multimethods randomized trial found that plain language versions improved adults understanding of health recommendations. J. Clin. Epidemiol..

[19] CDC (2024). Public Health Management of People with Suspected or Confirmed VHF or High-Risk Exposures. Viral Hemorrhagic Fevers (VHFs). https://www.cdc.gov/viral-hemorrhagic-fevers/php/public-health-strategy/people-with-suspected-or-confirmed-vhf-or-high-risk.html.

[20] CDC (2024). Guide for Clinicians Evaluating an Ill Person for VHF or Other High-Consequence Disease. Viral Hemorrhagic Fevers (VHFs). https://www.cdc.gov/viral-hemorrhagic-fevers/hcp/diagnosis-testing/evaluating-an-ill-person-for-vhf.html.

[21] CDC (2024). VHF Clinical Specimen Packaging and Shipping. Viral Hemorrhagic Fevers (VHFs). https://www.cdc.gov/viral-hemorrhagic-fevers/php/laboratories/specimen-packing.html.

[22] CDC (2024). Clinical Screening and Diagnosis for VHFs. Viral Hemorrhagic Fevers (VHFs). https://www.cdc.gov/viral-hemorrhagic-fevers/hcp/diagnosis-testing/index.html.

[23] CDC (2024). Laboratory Testing for Patients with a Suspected VHF or High-Consequence Disease. Viral Hemorrhagic Fevers (VHFs). https://www.cdc.gov/viral-hemorrhagic-fevers/php/laboratories/index.html.

[24] Infectious Disease Readiness-NYC Health. https://www.nyc.gov/site/doh/providers/emergency-prep/communicable-disease-preparedness.page.

[25] Meadows A.J., Stephenson N., Madhav N.K., Oppenheim B. (2023). Historical trends demonstrate a pattern of increasingly frequent and severe spillover events of high-consequence zoonotic viruses. BMJ Glob. Health.

[26] Smith K.F., Goldberg M., Rosenthal S., Carlson L., Chen J., Chen C., Ramachandran S. (2014). Global rise in human infectious disease outbreaks. J. R. Soc. Interface.

[27] Centers for Disease Control and Prevention, Assistant Secretary for Preparedness and Response Detailed Hospital Checklist for Ebola Preparedness. https://stacks.cdc.gov/view/cdc/25694.

[28] New York City Health + Hospitals Emergency Management. New York City Health + Hospitals Special Pathogens Program. Frontline Hospital Planning Guide: Special Pathogens. https://hhinternet.blob.core.windows.net/uploads/2019/07/NYCHH-Frontline-Hospital-Planning-Guide.pdf.

[29] Centers for Disease Control and Prevention, National Institute for Occupational Health and Safety About Hierarchy of Controls. https://www.cdc.gov/niosh/hierarchy-of-controls/about/index.html.

[30] New York State Department of Health New York City Health. NYS/NYC GUIDANCE FOR LABORATORY TESTING AND MANAGEMENT OF PERSONS-UNDER-INVESTIGATION, FOR EBOLA VIRUS DISEASE (EVD) IN NONDESIGNATED HOSPITALS. https://www.nyc.gov/assets/doh/downloads/pdf/labs/ebola-min-testing-capabilities.pdf.

